# Prognostic Factors Influencing Survival and a Treatment Pattern Analysis of Conventional Palliative Radiotherapy for Patients with Bone Metastases

**DOI:** 10.3390/curroncol28050331

**Published:** 2021-10-01

**Authors:** Patricia Ignat, Nicolae Todor, Radu-Mihai Ignat, Ofelia Șuteu

**Affiliations:** 1 Faculty of Medicine, Iuliu Hațieganu University of Medicine and Pharmacy, 400012 Cluj-Napoca, Romania; suteu.patricia@umfcluj.ro (P.I.); ofelia.suteu@iocn.ro (O.Ș.); 2 Prof. Dr. I. Chiricuță Oncology Institute, 400015 Cluj-Napoca, Romania; todor@iocn.ro

**Keywords:** bone metastases, conventional palliative radiotherapy, prognostic factors, overall survival, single fraction radiotherapy, multiple fraction radiotherapy

## Abstract

Background: Treatment indication for bone metastases is influenced by patient prognosis. Single-fraction radiotherapy (SFRT) was proven equally effective as multiple fractionation regimens (MFRT) but continues to be underused. Objective: Primary objectives: (a) to identify prognostic factors for overall survival and (b) to analyze treatment patterns of palliative radiotherapy (proportion of SFRT indication and predictive factors of radiotherapy regimen) for bone metastases. Methods: 582 patients with bone metastases who underwent conventional radiotherapy between January 1st 2014–31 December 2017 were analyzed. The Cox proportional hazard model was used to identify predictors of overall survival. For the treatment pattern analysis, 677 radiotherapy courses were evaluated. The logistic regression model was used to identify potential predictors of radiotherapy regimen. Results: The 3-year overall survival was 15%. Prognostic factors associated with poor overall survival were multiple bone metastases [hazard ratio (HR = 5.4)], poor performance status (HR = 1.5) and brain metastases (HR = 1.37). SFRT prescription increased from 41% in 2017 to 51% in 2017. Predictors of SFRT prescription were a poor performance status [odds ratio (OR = 0.55)], lung (OR = 0.49) and urologic primaries (OR = 0.33) and the half-body lower site of irradiation (OR = 0.59). Spinal metastases were more likely to receive MFRT (OR = 2.09). Conclusions: Based on the prognostic factors we identified, a selection protocol for patients candidates for palliative radiotherapy to bone metastases could be established, in order to further increase SFRT prescription in our institution.

## 1. Introduction

Bone metastases are one of the most frequent complications of advanced cancer. Breast, lung and prostate cancer are responsible for the majority of bone metastases [1]. It is estimated that they appear in two thirds of the patients with these malignancies, affecting either the pelvic bones, the spine, or limbs [2]. Bone metastases can lead to complications-fractures, hypercalcemia or spinal cord compression, affecting the performance status of patients and their quality of life [3]. When complications occur, like fractures of spinal cord compression, the surgical approach is chosen for either a palliative or curative purpose. In patients with reduced life expectancy, surgery may not represent a valid option, even in the context of complicated metastases [4].

Antalgic radiotherapy has a well-established role in managing patients with painful bone metastases; its efficiency has been proven in multiple research trials over the last decades [5]. It relieves pain, maintains bone functionality and integrity, with minimal adverse effects [6]. The efficacy of pain palliation is 60–85%, and the necessity of administrating pain relievers decreases [7]. Studies performed to date, among which multiple prospective studies and meta-analyses, showed no dose-response effect of radiotherapy, proving the equivalence between the single-8 Gy-fraction radiotherapy (SFRT) and the multiple fraction (MFRT) schedules, like 30 Gy in 10 fractions, 20 Gy in 5 fractions or other equivalences in patients with uncomplicated bone metastases. Evidence suggests that radiotherapy for bone metastases provides pain relief by inhibiting osteoclast-mediated bone reabsorption rather that by tumor cell kill, supporting the idea that high dose MFRT might not be necessary and that SFRT is enough for pain control [8]. Furthermore, no differences regarding acute gastrointestinal, hematologic, lung or central nervous system toxicities were reported between the two irradiation schedules [9,10,11,12]. An advantage of SFRT is the short duration of the treatment, hence hospitalization, contributing to the increase of patients’ life quality and the decrease of travelling costs as well as of costs incurred by the medical facility [13,14]. On the other hand, re-irradiation is more frequent after SFRT than after MFRT [15].

The survival of patients with bone metastases can vary from several months to a few years, therefore, in order to decide the optimal treatment strategy, it is helpful to use methods which help estimate prognosis [16]. The existing studies examining the potential prognostic factors included either only operated patients for bone metastases or only irradiated patients [17].

Although radiotherapy for bone metastases is a frequent treatment indication in any radiotherapy health service, choosing the optimal fractionation scheme can still be a challenge due to the clinical heterogeneity of the patient population. Moreover, although strong data sustain the advantages of SFRT, it is reluctantly being adopted in routine practice, for reasons varying from practitioners’ uncertainty regarding the relative benefits of SFRT [18] to financial aspects (some facilities receive funding based on the number of fractions performed) [19].

We performed a retrospective analysis of the clinical characteristics and treatment patterns in patients with bone metastases who underwent SFRT or MFRT at a tertiary cancer center. Our study had two primary objectives. First, we aimed to identify potential prognostic factors of overall survival, to help stratify patients with bone metastases in view of a more accurate treatment decision. Second, we aimed to assess treatment patterns for palliative radiotherapy of bone metastases in our institution, to establish if they are aligned with the most recent body of evidence. In this respect, we analyzed the prognostic factors associated with the indication of single versus multiple fraction radiotherapy, the proportion of SFRT indications out of the total irradiations for bone metastases. A secondary objective was to determine the re-treatment frequency after single fraction and multiple fraction radiotherapy.

## 2. Materials and Methods

### 2.1. Study Design and Patients

The medical records of 695 patients who underwent palliative radiotherapy for bone metastases at a tertiary cancer center, between 1 January 2014 and 31 December 2017, were anonymized and retrospectively reviewed. For the statistical analysis, we classified the radiotherapy regimens in either single fraction radiotherapy (SFRT) or multiple fraction radiotherapy (MFRT).

**Figure 1:**
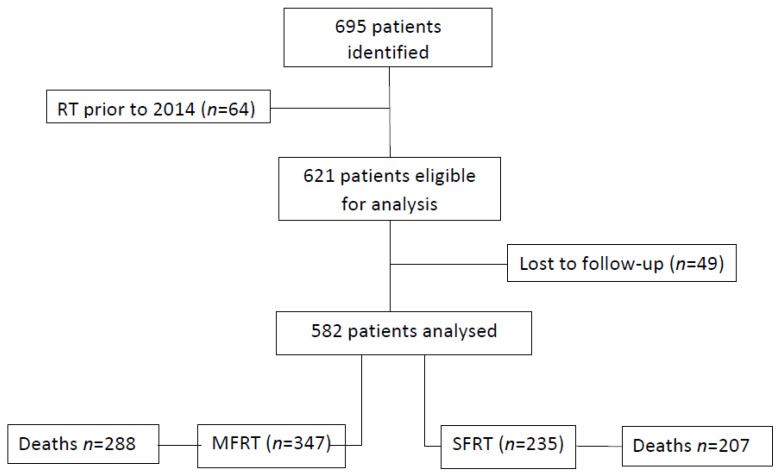
Study design. RT: Radiotherapy, MFRT: multiple-fraction radiotherapy, SFRT: single-fraction radiotherapy.

After excluding the patients with at least one radiotherapy course for bone metastases prior to 2014 (64 patients), and patients lost to follow-up (49 patients) a total of 582 patients remained in the study—347 patients in the MFRT group and 235 patients in the SFRT group (Figure 1). The patients included in the study underwent either a single irradiation sequence (498 patients) or several irradiation sequences on different anatomic regions (84 patients), in which case the first irradiation was considered for the OS analysis. For the treatment pattern analysis, we took into account the total number of radiotherapy courses performed, which was 677, considering the 84 patients who underwent multiple irradiations. In this way, for each radiotherapy course we could differentiate between several clinical characteristics that changed from one radiotherapy course to another for the same patient (e.g., age, PS, irradiated site).

### 2.2. Data Collection

#### 2.2.1. General Demographics

The data collected from the patients’ medical records were as follows: patient-related characteristics, including age at the time of radiotherapy, sex and Eastern Cooperative Oncology Group performance status (ECOG PS) score (4 categories from 1 to 4); tumor-related characteristics, including the type of primary tumor and control of primary; bone metastasis-related characteristics, including the number of bone metastases (categories: unique versus multiple), the presence of visceral metastases (lung, liver, brain), the site of bone metastases, the presence of complications (fracture or spinal cord compression), the irradiated anatomic region (axial skeleton, extremities).

The identification of bone metastases and their distribution was performed using Tc^99^ bone scintigraphy, CT scan and/or MRI. In addition, the evaluation of the primary tumor status and the presence of visceral metastases was performed by CT scan.

#### 2.2.2. Treatment Related Information

The information related to the treatment were acquired from the radiotherapy records: the number of fractions, total dose, the irradiated anatomic region, re-irradiations. In addition, in patients who underwent multiple irradiation sequences for metastases in different skeletal areas, we recorded all the information regarding each treatment performed.

In painful or complicated bone metastases, the decision for treatment was taken in multidisciplinary institutional committees. Patients with fractures or spinal cord compression, with or without neurological deficit and a life expectancy of over two months, were proposed for orthopedic surgical or neurosurgical procedures (50 patients). Palliative radiotherapy was performed both in patients with surgical indication (post-operative) and in patients who did not need surgery.

Radiotherapy was performed on a 6 MV linear accelerator. 91% of irradiations were 2D, and 9% were 3D conformal irradiations. The irradiation regimen consisted either of a single 8 Gy fraction or a multiple-fraction version (30 Gy in 10 fractions, 20 Gy in 4 or 5 fractions). During a course of radiotherapy, the target volumes were either a single anatomic site (the spine, for example) or more sites when half-body lower irradiation was performed (the target volume includes the lumbar spine, the pelvis and the proximal third of both femurs). The choice of the irradiation regimen was at the latitude of the treating physician.

### 2.3. Follow-Up and Outcomes

The median follow-up of patients still alive at the end of the study was 42 months (range 18.5–74.7 months). The overall survival was calculated from the date the palliative radiotherapy was initiated until the date of death from any cause or, if death did not occur, until the end of the study (31 December 2020).

### 2.4. Statistical Analysis

#### Analysis of Overall Survival (OS)

The 1-, 2- and 3-year overall survival was determined through the Kaplan-Meier method. Survival differences between the groups were evaluated through the log-rank test.

A Cox proportional hazard model with hazard ratios (HRs) and 95% confidence intervals (CIs) was used to evaluate the impact of prognostic factors on OS. In multivariate analysis, we included the prognostic factors significantly associated with OS in univariate analysis.

### 2.5. Treatment Pattern Analysis

We analyzed possible factors associated with the indication of SFRT or MFRT. We used the chi-squared test to identify the differences between baseline characteristics by fractionation schedule. Then, the difference of averages between the two groups of variables was tested through the analysis of variance (ANOVA). The variables identified as potential predictors of statistical significance were included in the uni- and multivariate analysis—the logistic regression model, calculating the Odds Ratio values and the 95% confidence interval.

All the statistical tests used were considered statistically significant at a *p*-value of <0.05.

For the statistical analysis, we used Excel and version 17 SPSS packages (SPSS Inc., Chicago, IL, USA).

## 3. Results

### 3.1. Patient Characteristics

The study group consisted of 582 patients who underwent 677 palliative radiotherapy courses on 829 bone metastatic sites

Patient characteristics are presented in Table 1. The median age was 60 years (range 18–86). The most frequent primary tumor was lung cancer (34%, 197/582), followed by breast cancer (31%, 179/582) and urogenital cancers (17%, 102/582). The remaining primary tumors were digestive and gynecological cancers or other rare localizations (melanomas, sarcomas, head and neck and thyroid cancers). Half of the patients (51%, 297/582) presented bone-only metastases, whereas the rest of the patients (285/582) also had visceral metastases in one, two or more sites (lung, liver, brain, etc.). Complicated bone metastases (fractures, spinal cord compression) occurred in 55% (172/582) patients. In the time interval considered, 85.5% (498/582) of the patients benefited from a single radiotherapy treatment course, whereas the rest of the patients (84/582) underwent two, three or four irradiations on different anatomical regions.

**Figure 2:**
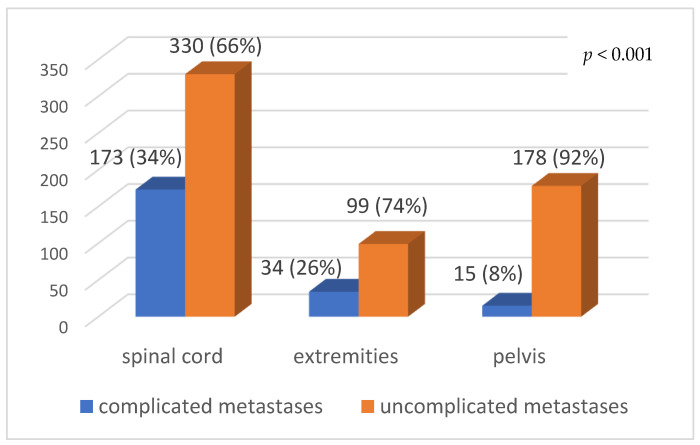
Complications according to the site of metastases.

The frequency of complications differed depending on the metastatic site. Spinal metastases were complicated in 34% (173/503) of cases, whereas pelvic lesions had only 8% (178/193) complications (*p* < 0.001) (Figure 2).

### 3.2. Overall Survival and Its Association with Clinical Features

**Figure 3:**
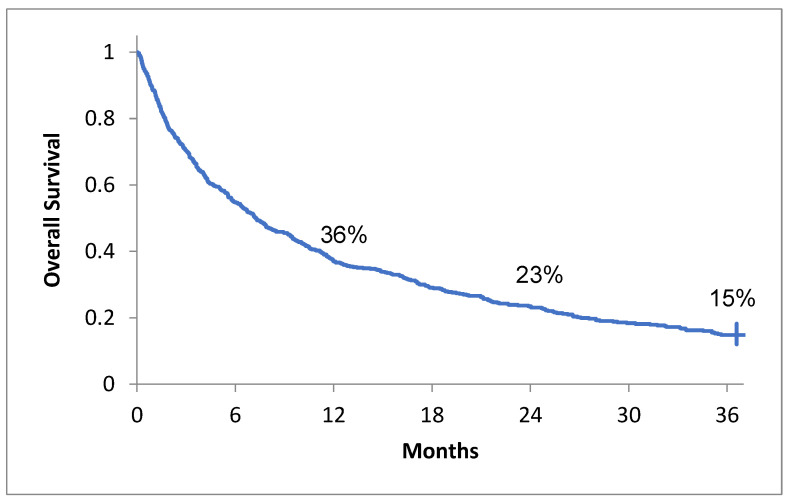
1- 2- and 3-year OS of the study group.

At the end of the study there were 87 patients alive, 59 in the MFRT group and 28 in the SFRT group. The 1-, 2- and 3-year OS was 36% (95% CI [33–41%]), 23% (95% CI [20–27%]), and 15% (95% CI [12–18%]), respectively (Figure 3). The median survival was 7.3 months (95% CI [6.3–8.1]).

After analyzing the association between the clinical/tumoral features and OS, we observed that the following variables significantly influence the 3-year OS: age, sex, ECOG PS, type of primary tumor, control of the primary tumor, presence of visceral metastases, particularly brain metastases, the number of bone metastases, and complications of bone metastases (Table 2). Higher 3-year OS rates were observed in women compared to men (23%, 95%CI [19–29%] vs. 6%, 95%CI [4–10%], *p* < 0.001) and in patients aged ≤60 years compared to older (17%, 95%CI [13–22%], vs.13%, 95%CI [9–17%], *p* = 0.2). The longest 3-year OS was identified in patients with breast cancer (32%, 95%CI [25–39%]) and the shortest OS in patients with lung cancer (3%, 95%CI [1–7%]), *p* < 0.001.

For patients with ECOG PS of 1, the 3-year OS rate was 24% (95%CI [18–30%]), decreasing to 13% (95%CI [9–19%]) for ECOG 2, 9% (95%CI [5–15%]) for ECOG 3, and none of the patients with PS of 4 survived at 3 years (*p* < 0.001). The 3-year OS rate for patients with one bone metastasis was 72% (95%CI [55–85%]), versus 11% (95%CI [8–14%]) for patients with multiple bone metastases (*p* < 0.001).

### 3.3. Multivariate Analysis

For the multivariate analysis, the factors which significantly influenced the 3-year OS were the type of primary tumor, ECOG PS, control of the primary tumor, the number of bone metastases and the presence of brain metastases (Table 3). The most important factors significantly associated with a low survival risk were the presence of multiple bone metastases (HR = 5.4, 95%CI [2.94–9.91], *p* < 0.001), a decreased performance status (HR = 1.5, 95%CI [1.38–1.69], *p* < 0.001) and the presence of brain metastases (HR = 1.37, 95%CI [1.08–1.73], *p* < 0.001). Other prognostic factors for poor survival were primary tumor other than breast (HR = 1.24, 95%CI [1.05–1.77], *p* < 0.001), no control of the primary tumor (HR = 1.26, 95%CI [1.04–1.52], *p* < 0.001) and complications of bone metastases (HR = 1.2, 95%CI [1.09–1.44], *p* = 0.04).

### 3.4. Treatment Pattern Analysis

**Figure 4:**
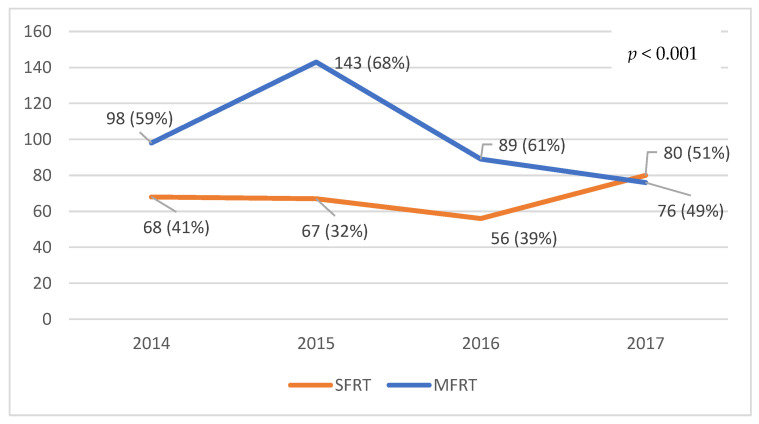
Radiotherapy regimens prescribed each year, in the period 2014–2017.

A total of 677 irradiations were performed on the 582 patients included in the study. The overall proportion of SFRT between 2014 and 2017 was 40% (271/677). The ratio of SFRT irradiation varied in the analyzed time interval (Figure 4). There was an increase of SFRT prescription from 41% (68/166) in 2014 to 51% (80/150) in 2017 (*p* < 0.001). In the 84 patients undergoing multiple irradiations on different sites, if the first irradiation was MFRT, the second one was more likely to be MFRT as well: 73% (39/53 patients) compared to 27% (14/53 patients) SFRT. Moreover, of the patients who initially received SFRT, 52% (16/31 patients) received MFRT at their second irradiation (*p* = 0.04).

MFRT was prescribed more than SFRT in both age groups studied—63% in patients ≤60 years (227/358 patients) and 56% in patients >60 years (179/319 patients) and respectively in both sexes—66% in women (227/343) and 54% in men (179/334) (Table 4). A high frequency of SFRT indication was observed in urogenital cancers—51% (62/121 patients); for all other sites, MFRT was predominant (*p* < 0.001). The rate of SFRT increased with the decrease of the patients’ performance status: 76% of patients (173/228) with a PS of 1 underwent MFRT and only 24% (55/228) benefited from SFRT, whereas patients with a PS of 4 received SFRT in a higher proportion: 56% (23/41 patients) versus 44% (18/41 patients) MFRT (*p* < 0.001). Most frequently, SFRT was indicated for patients who underwent half-body lower radiotherapy—59% (71/121 patients), whereas, for spinal metastases, SFRT was less prescribed—32% (124/382 patients) (*p* < 0.001).

On multivariate analysis, urogenital and lung tumors (OR = 0.33, 95%CI [0.21–0.53], respectively OR = 0.49, 95%CI [0.33–0.71], *p* < 0.001) are least likely to benefit from MFRT. In addition, a poor performance status (OR = 0.55, 95%CI [0.45–0.66], *p* < 0.001), and the half-body lower irradiation (OR = 0.59, 95%CI [0.36–0.99], *p* = 0.04) decrease the chances for MFRT. In contrast, the presence of spinal bone metastases increases the chances of MFRT irradiation (OR = 2.1, 95%CI [1.41–3.12], *p* < 0.001) (Table 5).

There were 54 re-irradiations in the studied period, 60% (32/52 patients) of which were performed after an initial SFRT. In this respect, the re-treatment frequency was 0.5% after MFRT and 12% after SFRT (*p* < 0.001).

## 4. Discussion

A frequent complication of advanced cancer, bone metastases can cause excess morbidity, therefore the primary role of bone metastases treatment is to relieve symptomatology and prevent skeletal-related events [20]. Treatment options are numerous, therefore estimating the prognosis of patients with bone metastases can facilitate the choice of treatment. For patients undergoing palliative radiotherapy, although recent years brought standardization of treatment, irradiation schedule is sometimes indicated following subjective criteria.

From our results, the 3-year OS of patients with bone metastases is poor (15%). It is influenced by the performance status of patients, the presence of complicated and multiple bone metastases and by the subsequent occurrence of brain metastases. Regarding radiotherapy fractionation, in our institution, SFRT prescription was 40% of the total palliative radiotherapy courses in the interval 2014–2017 and was highest in 2017 (50%). SFRT was more likely to be prescribed in urogenital and lung tumors, patients with poor performance status and for the half-body lower sites. The re-treatment frequency was 12% after SFRT and 0.5% after MFRT.

We acknowledge our study’s limitations issued mainly from its retrospective design. With respect to the OS analysis, we were unable to test more variables with possible prognostic value because they were inconsistently available in the patients’ medical records, such as biochemical markers (e.g., calcium and alkaline phosphatase levels, albumin levels, leucocyte count), previous systemic therapies (many patients were previously treated in other facilities and had incomplete documentation), molecular subtype for breast and lung cancers, comorbidities. Any number of these variables would have contributed to a more sensitive prognostic tool of OS. Regarding the treatment pattern analysis, the most notable downfall of our study is the lack of data on pain response and local control after SFRT versus MFRT, which would have put into perspective our results on re-treatment frequency. The retrospective nature of the study could arguably be a source of patient selection bias. Nonetheless, it was not the purpose of the study to compare SFRT with MFRT in terms of efficiency, the re-treatment frequency being a secondary objective, but to analyze exactly the factors prognostic to the choice of fractionation schedule. In addition, considering that re-treatment is mainly due to pain relapse, selection bias has little influence on patients’ subsequent perception of pain.

The median survival in our group of patients was 7.3 months, similar to Willeumier et al. [21], but far lower than the survival reported by other authors—14 months [22]. On the other hand, we obtained similar results to other studies regarding OS rates. In this respect, Katagiri et al. reported identical OS rates with the ones we obtained 36%, 23% and 16% [4], whereas Kubota et al. obtained lower OS rates at one and two years: 23% and 13% [23]. The survival differences between the studies can be attributed to a different selection of patients and their variable clinical characteristics.

For practitioners treating bone metastases patients it is important to determine their prognosis, in order to select patients who are candidates for surgery. Moreover, the prognosis of patients can influence the decision of fractionation regimen in patients who undergo radiotherapy. For these reasons, there were multiple initiatives for developing scores based on prognostic factors associated with survival with bone metastases [24,25,26,27,28] and prognosis prediction models based on machine-learning [29,30,31] (Table 6).

Similar to us, the performance status of the patients and the presence of single versus multiple bone metastases was found to influence prognosis by multiple authors [21,26,27,28]. Contrarily, other authors did not prove that multiple bone metastases would affect the prognosis of patients [23]. In our study we found no influence of visceral metastases on OS, which is in contrast with other published results [29,30]. Nevertheless, when we isolated only the cases of brain metastases, we noticed that these negatively impact the patients’ survival. This was also reported by Willeumier et al. [21]. Moreover, our analysis shows that complications like spinal cord compression or pathological fractures worsen the prognosis of patients, which is concordant with other published studies [4,27].

In our analysis, the primary tumor type seems to influence the prognosis, with breast cancer having the best prognosis, compared to all other sites. In a retrospective study comprising 125 patients, Zhang et al. proved that the tumor type influences survival, with significantly reduced survival rates for colorectal and esophageal cancer [22]. Other authors who analyzed larger patient series reported more specific differences, after grouping different primaries into categories of either favorable/moderate/unfavorable tumor profiles [21,28] or fast/moderate/slow growing tumors [27].

An aspect worth mentioning is the significant difference in OS between men and women, observed on univariate analysis, although no longer significant on multivariate analysis. Sex differences in OS, favoring women have been reported in patients with esophageal and rectal cancers treated with radiotherapy [32,33]. Possible explanations are that variances in endocrinology, metabolism, immunity and tumor suppression between sexes are affecting how radiotherapy influences not only OS but also acute and long-term side effects [34,35].The choice of the irradiation regimen in patients with bone metastases depends on the purpose of the irradiation and the patients’ prognosis. SFRT for pain relief is recommended to patients with a life expectancy under six months [14], also having the advantage of lower hospitalization costs, whereas offering the same antalgic efficiency as MFRT regimens [36]. Furthermore, SFRT is recommended in cases with no complications, such as fractures or spinal cord compression [37]. However, despite all its advantages, SFRT continues to be less recommended than MFRT. For example, a 2013 study that analyzed the pattern of antalgic irradiation indication for bone metastases in patients suffering from prostate cancer in the USA concluded that SFRT was prescribed in only 3% of cases. In contrast, MFRT with over ten fractions was indicated for more than 50% of patients [38]. The possible explanations for the large variability when indicating a certain irradiation schedule could be the individual interpretation of practice guidelines, personal, professional practice and the routine of colleagues, but also financial motivations (the remuneration of radiotherapy services depending on the number of fractions performed) [2] (Table 7).

In our study, we observed that between the first and last year (2014 and 2017 respectively), the frequency of SFRT indication increased by 10%, from 41% to 51%, figures similar to those communicated by other authors [2], but superior to other studies, mainly from the USA, which report SFRT indication rates of up to 13% [42,47,48]. Furthermore, recent results from Kim et al. showed that SFRT prescription did not increase even after an active campaign to disseminate guidelines favoring SFRT among practitioners [46]. On the other hand, Thavarajah et al. reported a proportion of SFRT irradiations as high as 65% since 2005, which was maintained throughout the years [44].

Our analysis suggested that the prescription of radiotherapy schedules was associated with performance status. The rate of SFRT prescription increased with decreasing PS. Since our results indicate that the PS influences the prognosis of patients, we can conclude that the choice of the irradiation regimens in our institution was made in line with the international recommendations of prescribing the irradiation regimen depending on the life expectancy of patients [37,49]. Similar results were reported by Olson et al. [2], whereas other studies did not find an association between the PS of patients and the fractionation schedule [46].

When analyzing the SFRT prescription proportion according to the primary tumor type, we observed a 51% indication in patients with urogenital cancers, mostly prostate cancer. Even the multivariate analysis suggested that the prescription of SFRT is significantly associated with urogenital cancers. The result is similar to other studies, which reported a 56% proportion, although, some authors reported only 3% SFRT prescriptions in these patients [38].

The site of bone metastases significantly influences the choice of radiotherapy regimens. We observed that the presence of spinal metastases favors the choice of MFRT, whereas the irradiation of the half-body lower region is performed using most likely SFRT. The predilection for MFRT in spine irradiation was reported by other authors as well [2,46].

After a three-year follow-up, the re-irradiation frequency was 0.5% after MFRT and almost 12% after SFRT. Out of the total of 54 re-irradiations identified during the period of the study, 60% were prescribed after initial SFRT irradiation. These results are comparable to those in the literature, at least as to the post-SFRT re-irradiation frequency, with values of 15–20%, whereas the post-MFRT re-irradiation frequency varies between 5% and 8% [7,50,51]. The literature mentions higher re-irradiation frequency in patients with longer survival and who underwent SFRT, compared to MFRT, even if a 2012 study concluded that the durability of the antalgic response after SFRT does not differ in the case of long-term survivors compared to the patients with a more reserved prognosis [52]. The superiority of MFRT in decreasing re-treatment necessity could be real, or only apparent, as a reflection of patient choice or clinical practice, where physicians are more reluctant to retreat after a higher dose MFRT, especially spinal metastases, for fear of toxicity. [15,53].

More recently, advances in the conformality of image-guided radiotherapy techniques, such as stereotactic body radiotherapy (SBRT) have enabled the delivery of higher radiation doses, with better sparing of healthy tissues. Multiple studies suggested that the delivery of ablative doses improves pain response and duration of pain control, alongside better local control [54,55,56,57]. Two phase II randomized trials comparing SBRT with MFRT using conventional 2D, 3D radiotherapy or intensity modulated radiotherapy (IMRT) concluded that SBRT was associated with quicker and more consistent pain improvement as well as higher local control rates [58,59]. A phase III randomized trial addressing the role of SBRT by IMRT with simultaneously integrated boost compared to conventional MFRT in patients with spinal metastases is ongoing, with overall pain reduction rate as primary endpoint and retreatment rates, local control and OS as secondary endpoints [60]. Further studies are required in this field that offers promising perspectives, regarding optimal doses, fractionation, and response assessment [55].

Our study’s strengths rely on the large number of patients included, representative for heterogeneous real-world patient populations, considering they were treated in a tertiary oncology center, which has nationwide addressability. Few data exist in the literature in our country about clinical characteristics and palliative radiotherapy treatment patterns in patients with bone metastases. To our knowledge, this is the most comprehensive examination of the kind. Therefore, we consider that this analysis can be considered as a selection tool for patients with bone metastases candidates for palliative radiotherapy, having the advantage that the prognostic factors investigated can be easily obtained during the initial diagnostic assessments of patients.

## 5. Conclusions

Overall survival with bone metastases is poor. Patients with favorable outcome (good performance status, single bone metastasis and no brain metastases) can benefit the most from multiple-fraction regimens, which are less likely to require reirradiation. Single-fraction radiotherapy remains a valid choice for patients with a more reserved outcome. Single-fraction radiotherapy is increasingly being prescribed at our institution, but additional steps could be taken to further increase it, such as to establish a selection protocol for patients candidates for palliative radiotherapy to bone metastases based on the prognostic factors we identified.

## Figures and Tables

**Table 1:** Clinical characteristics of patients.

Characteristics	No. of Patients	%
Sex		
female	286	49%
male	296	51%
Age		
≤60 years	298	51%
>60 years	284	49%
ECOG performance status		
0–1	203	35%
2	196	33%
3	149	26%
4	34	6%
Primary tumor		
lung	197	34%
digestive	47	8%
gynecologic	21	4%
breast	179	31%
urogenital	102	17%
Other sites	36	6%
Control of primary tumor		
Yes	240	41%
No	342	59%
No. of visceral metastases sites		
No visceral metastases	297	51%
1	160	27%
2	86	15%
≥3	39	7%
Lung metastases		
Yes	115	20%
No	467	80%
Brain metastases		
Yes	89	15%
No	493	85%
Liver metastases		
Yes	141	24%
No	441	76%
Complicated bone metastases		
Yes	172	30%
No	410	70%
Bone metastases surgery		
Yes	50	9%
No	532	91%
No. of irradiations for bone metastases		
1	498	85.5%
2	75	13%
3	7	1.20%
4	2	0.3%
Irradiation schedule		
MFRT	347	60%
SFRT	235	40%
Total	582	100%

Note: for the 84 patients who underwent multiple irradiations on different regions, only the first was taken into account for the OS analysis.

**Table 2:** Univariate analysis of 3-year overall survival.

Variable		3-Year OS (%)	95% CI	*p*-Value
Age	≤60 years (298)	17%	12–22%	0.02
	>60 years (284)	13%	9–17%	
Sex	female (286)	23%	19–29%	<0.001
	male (296)	6%	4–10%	
ECOG performance status	1 (203)	24%	18–30%	<0.001
	2 (196)	13%	9–19%	
	3 (149)	9%	5–15%	
	4 (34)	0%	-	
Primary tumor	lung (197)	3%	1–7%	<0.001
	breast (179)	32%	25–39%	
	urogenital (102)	14%	8–23%	
	digestive (47)	6%	2–17%	
	gynecologic (21)	19%	8–40%	
	other sites (36)	3%	0.6–17%	
No. of bone metastases	single (34)	72%	55–85%	<0.001
	multiple (548)	11%	8–14%	
Control of primary tumor	yes (240)	24%	19–31%	<0.001
	no (342)	8%	5–12%	
Visceral metastases	yes (285)	7%	5–11%	<0.001
	no (297)	22%	18–28%	
Brain metastases	yes (89)	2%	0.02–8%	<0.001
	no (493)	17%	14–21%	
Lung metastases	yes (115)	8%	4–16%	0.10
	no (467)	16%	13–20%	
Liver metastases	yes (141)	9%	5–15%	0.11
	no (441)	17%	13–21%	
Complications of bone metastases	yes (222)	13%	9–19%	0.04
	no (360)	16%	12–20%	
Irradiated anatomical region	Axial skeleton (spine + pelvis) (529)	16%	8–29%	0.57
	extremities(53)	15%	12–18%	
Irradiation scheme	MFRT(347)	17%	12–21%	<0.001
	SFRT(235)	12%	8–17%	

**Table 3:** Multivariate analysis of 3-year overall survival.

Variable	HR	95% CI	*p*-Value
Age (continuous variable)	1.00	0.99–1.01	0.31
Sex (female vs. male)	1.19	0.9–1.49	0.11
Primary tumor (breast versus the rest of sites)	1.24	1.05–1.77	<0.001
ECOG performance status (reference PS = 1)	1.53	1.38–1.69	<0.001
No. of bone metastases (single vs. multiple)	5.40	2.94–9.91	<0.001
Complications associated to bone metastases (no vs. yes)	1.20	1.09–1.44	0.04
Control of primary tumor (yes vs. no)	1.26	1.04–1.52	0.001
Visceral metastases (no vs. yes)	1.16	0.95–1.41	0.12
Brain metastases (no vs. yes)	1.37	1.08–1.73	<0.001

**Table 4:** The association of prescription of radiotherapy regimens based on patient characteristics.

Characteristics		Irradiation Schedule			
		MFRT	SFRT	Total	*p*-Value
Age	≤60 years	227 (63%)	131 (37%)	358	<0.05
	>60 years	179 (56%)	140 (44%)	319	
Sex	female	227 (66%)	116 (34%)	343	<0.001
	male	179 (54%)	155 (46%)	334	
Primary tumor	lung	118 (54%)	99 (46%)	217	<0.001
	gastrointestinal	36 (68%)	17 (32%)	53	
	gynecologic	16 (73%)	6 (27%)	22	
	breast	153 (68%)	72 (32%)	225	
	urogenital	59 (49%)	62 (51%)	121	
	other sites	24 (62%)	15 (38%)	39	
ECOG performance index	1	173 (76%)	55 (24%)	228	<0.001
	2	138 (57%)	104 (43%)	242	
	3	77 (46%)	89 (54%)	166	
	4	18 (44%)	23 (56%)	41	
Control of primary tumor	yes	203 (62%)	127 (38%)	330	0.42
	no	203 (58%)	144 (42%)	347	
Visceral metastases	yes	229 (60%)	150 (40%)	379	0.79
	no	177 (59%)	121 (41%)	298	
Irradiated anatomical site	spine	258 (68%)	124 (32%)	382	<0.001
	pelvis	41 (57%)	31 (43%)	72	
	half-body lower	50 (41%)	71 (59%)	121	
	extremities	57 (56%)	45 (44%)	102	
Complications associated to metastases	yes	139 (63%)	83 (37%)	222	0.33
	no	267 (59%)	188 (41%)	455	
Re-irradiation	yes	22 (41%)	32 (59%)	54	<0.001
	no	384 (62%)	239 (38%)	623	
Total		406 (60%)	271 (40%)	677	

**Table 5:** Multivariate regression of factors influencing the choice of the irradiation scheme.

Variable	Odds Ratio in Favor of MFRT	95% CI	*p*-Value
Sex (female vs. male)	0.73	0.47–1.13	0.16
Age (≤60 years vs. >60 years)	0.83	0.59–1.17	0.29
Primary site (urogenital vs. other sites)	0.33	0.21–0.53	<0.001
Primary tumor (lung vs. other sites)	0.49	0.33–0.71	<0.001
ECOG performance status (reference PS = 1)	0.55	0.45–0.66	<0.001
Irradiated site (spine vs. other sites)	2.09	1.41–3.12	<0.001
Irradiated site (half-body lower vs. other sites)	0.59	0.36–0.99	0.04

**Table 6:** Studies assessing prognostic factors associated with overall survival with bone metastases.

First Author (Year)	Patient Population	Negative Prognostic Factors of Survival	Survival	Reference
Katagiri (2005)	350 patients with bone metastases irradiated and/or operated	Type of primary (lung, stomach, liver, poor PS, visceral/brain metastases, previous chemotherapy, multiple bone metastases	1-year OS 48% 2-year OS 33% 3-year OS 23%	[26]
Mizumoto (2008)	544 patients with spinal metastases	Increasing age, poor PS, unfavorable primary, visceral metastases, multiple bone metastases, previous chemotherapy, serum calcium, neurologic deficit	Median OS 5.9 months 1-year OS 32% 2-year OS 19%	[25]
Janssen (2015)	927 patients operated for long bone metastases	Age, comorbidities, increased BMI, tumor type with poor prognosis, multiple bone metastases, visceral metastases, low hemoglobin levels	Median OS 8.6 months	[28]
Zhang (2016)	125 patients with bone metastases irradiated and/or operated	Sex, PS, primary tumor (esophagus, colorectal), T stage, differentiation	Median OS 14.1 months	[22]
Willeumier (2018)	1520 irradiated patients with long bone metastases	Poor PS, visceral/brain metastases, unfavorable clinical profile (lung, colon, esophagus, melanoma, stomach, liver)	Median OS 7.4 months	[21]

**Table 7:** Patterns of practice regarding irradiation regimens in bone metastases.

First Author (Year)	Time Period	No. of RT Courses	% of SFRT Prescriptions	Factors Associated with SFRT	Reference
Szostakiewicz (2004)	1995–2002	1754	19%	Lung and breast primaries, irradiation of ribs and long bones	[39]
Haddad (2005)	1998–2002	882	32%	Increased age, poor PS, greater weight loss	[40]
Bradley (2008)	1999–2005	965	65%	Increased age, prostate primaries, poor PS, non-spine sites	[41]
Beriwal (2012)	2003–2010	7905	3.9%	Spine and extremities were more likely to receive MFRT	[42]
Bekelman (2013)	2006–2009	3050	3.3%	Poor PS	[38]
Laugsand (2013)	1997–2007	14380	31.3%	Increased age, poor PS, lung and prostate primaries	[43]
Thavarajah (2013)	2005–2012	2549	65%	Increased age, poor PS, prostate primary, non-spine sites, re-irradiation	[44]
Olson (2014)	2007–2011	16898	49.2%	Hematologic and prostate primaries, irradiation of ribs and extremities, poor PS	[2]
Ashworth (2016)	1984–2012	161835	43.9%	Increased age, poor PS, non-spine sites	[45]
Kim (2020)	2016	807	62%	Prostate primary, uncomplicated metastases, non-spine sites, re-irradiation	[46]

## Data Availability

Data are unavailable due to patient confidentiality.
